# Elevated metabolic rate and skeletal muscle oxidative metabolism contribute to the reduced susceptibility of NF‐*κ*B p50 null mice to obesity

**DOI:** 10.14814/phy2.13836

**Published:** 2018-09-25

**Authors:** Bankim A. Bhatt, Nikolaos Dedousis, Ian J. Sipula, Robert M. O'Doherty

**Affiliations:** ^1^ Department of Medicine Division of Endocrinology and Metabolism University of Pittsburgh Pittsburgh Pennsylvania; ^2^ Department of Microbiology and Molecular Genetics University of Pittsburgh Pittsburgh Pennsylvania

**Keywords:** Inflammation, metabolic rate, NF‐*κ*B p50, obesity, oxidative metabolism, skeletal muscle

## Abstract

Mice with a deletion of the p50 subunit of the proinflammatory nuclear factor kappa B pathway (NF‐*κ*B p50) have reduced weight compared to wild‐type control mice. However, the physiological underpinning of this phenotype remains unknown. This study addressed this issue. Compared to littermate controls, lean male p50 null mice (p50^−/−^) had an increased metabolic rate (~20%) that was associated with increased skeletal muscle (SkM, ~35%), but not liver, oxidative metabolism. These metabolic alterations were accompanied by decreases in adiposity, and tissue and plasma triglyceride levels (all ~30%). Notably, there was a marked decrease in skeletal muscle, but not liver, DGAT2 gene expression (~70%), but a surprising reduction in muscle PPAR *α* and CPT1 (both ~20%) gene expression. Exposure to a high‐fat diet accentuated the diminished adiposity of p50^−/−^ mice despite elevated caloric intake, whereas plasma triglycerides and free fatty acids (both ~30%), and liver (~40%) and SkM (~50%) triglyceride accumulation were again reduced compared to WT. Although SkM cytokine expression (IL‐6 and TNF *α*, each ~100%) were increased in p50^−/−^ mice, neither cytokine acutely increased SkM oxidative metabolism. We conclude that the reduced susceptibility to diet‐induced obesity and dyslipidemia in p50^−/−^ mice results from an increase in metabolic rate, which is associated with elevated skeletal muscle oxidative metabolism and decreased DGAT2 expression.

## Introduction

Altered activity of the prototypical proinflammatory NF‐*κ*B pathway (Collart et al. 1990; Shakhov et al. 1990; Pahl 1999; Tak and Firestein 2001; Ghosh and Karin 2002; Li and Verma 2002; Weigert et al. 2004; Jove et al. 2005, 2006) has been implicated in altering insulin sensitivity, lipid homeostasis, and adiposity, suggesting a role for NF‐*κ*B in the pathogenesis of metabolic abnormalities of obesity (Kim et al. 2001; Yuan et al. 2001; Hundal et al. 2002; Sinha et al. 2004; Arkan et al. 2005; Cai et al. 2005; Bhatt et al. 2006). Studies of note have demonstrated that inhibition or heterozygous deletion of IKK*β*, an upstream regulator of the NF‐*κ*B pathway, improves the abnormal metabolic sequalae of obesity (Yuan et al. 2001; Arkan et al. 2005; Cai et al. 2005). Furthermore, in humans, mice and cell culture, the administration of salicylate, a pharmacological inhibitor of IKK*β*, an upstream regulator of the NF‐*κ*B pathway, improves the insulin resistance associated with obesity, type II diabetes, and lipid oversupply (Kim et al. 2001; Yuan et al. 2001; Hundal et al. 2002; Sinha et al. 2004). Despite these studies, the metabolic effects of manipulation of elements of the NF‐*κ*B pathway other then IKK*β* are less well understood. In one study (Gao et al. 2009), it was reported that deletion of the p50 subunit of NF‐*κ*B decreases hepatic insulin resistance associated with diet‐induced obesity. However, reduced weight gain on a high‐fat diet was also evident in these animals, and the physiological basis of this phenotype was not addressed. The purpose of this study was to address this issue. The data demonstrate that a major cause of resistance to weight gain in p50 null mice is an increased metabolic rate that is associated with elevated skeletal muscle oxidative metabolism and markedly decreased expression of skeletal muscle DGAT2.

## Materials and Methods

### Ethics statement

All procedures in mice were approved by the Institutional Animal Care and Use Committee (IACUC) of the University of Pittsburgh (protocol approval number 0711702), and were in accordance with the National Research Council's *Guide for the Care and Use of Laboratory Animals*. NF‐*κ*B p50 null mice (Sha et al. 1995) were purchased from Jackson Laboratories (B6;129P‐*Nfkb1* ^*tm1Bal*^ */*J, Bar Harbor, ME) at an age of 10–12 weeks, housed at room temperature in an AALAC approved pathogen free facility, maintained on a constant 12:12‐h light–dark cycle, and given free access to food and water. P50^−/−^ mice were bred with C57BL/6J mice to produce N1‐F1 heterozygous mice, which were then intercrossed to produce N1‐F2 p50 null and their wild‐type (WT) littermate controls. Only male mice were used in studies.

### Diets and measurements of body weight, food intake

Male mice were ad libitum fed either a standard rat chow (11% of calories from fat) or a high‐fat diet (45% of calories from fat, Harlan Teklad, Madison WI. TD 96001) for up to 24 weeks. During this period body weight and food intake were monitored biweekly. At the completion of the diet period, animals were fasted overnight, anesthetized with pentobarbital and blood (~1 mL), skeletal muscle [gastrocnemius], liver and adipose tissue [perirenal and epidydimal] were extracted, weighed, and flash frozen in liquid nitrogen.

### Metabolic rate and activity measurements

Locomotor activity of p50 null and wild‐type mice was determined using a 48 channel open field test chamber (Med Associates, Inc. Georgia, Vermont) between 6 am and 8 am in a quiet room under dimmed light, using software supplied by the manufacturer. Activity was measured for 30 min, using cumulative data from fifteen 2‐minute intervals. Metabolic rate was measured at room temperature using open‐circuit indirect calorimetry in a small animal single chamber Oxymax system (Columbus Instruments, Columbus, OH). Animals were allowed to adapt to the chamber after which data were collected every 30 sec for 22 h. For analysis, the cumulative data for each hour were used.

### Tissue and plasma measurements

Tissue triglycerides were measured as previously described (Huang et al. 2006). Plasma triglycerides were assessed using the Infinity triglyceride kit with Lintrol lipids as standards (Sigma, St. Louis, MO). Free fatty acids were measured using the free fatty acid, half‐micro test kit (Roche Diagnostics, Penzberg, Germany). Plasma TNF*α*, IL‐6 (R&D Systems, Minneapolis, MN) insulin, adiponectin, leptin, and resistin (Linco Research, St. Charles, MO) were measured using ELISA kits.

### Fatty acid oxidation

Fatty acid oxidation was assessed as previously described (Perdomo et al. 2004; Huang et al. 2006). Briefly, soleus muscle and liver slices from p50^−/−^ and WT littermate controls were extracted and incubated in KRH buffer (118.4 mmol L^−1^ NaCl, 1.19 mmol L^−1^ KH_2_PO_4_, 4.76 mmol L^−1^ KCl, 1.19 mmol L^−1^ MgSO_4_, 24.9 mmol L^−1^ NaHCO_3_, 1.2 mmol L^−1^ CaCl_2_·H_2_0) containing 5 mmol L^−1^ dextrose and 0.4 mmol L^−1^ palmitate for 20 min at 29°C. Then, 3‐[^3^H]‐palmitate (Perkin Elmer, Boston, MA) was added to a final concentration of 2 *μ*Ci/mL and the tissues were incubated for 60 min at 29°C, and the incubation buffer was then collected for determination of tritiated water production using the vapor‐phase equilibration method, as previously described (Huang et al. 2006). For experiments utilizing IL‐6 and TNF*α*, solei muscles were exposed to the cytokines (10 ng/mL) for 1‐h prior to addition of 3‐[^3^H]‐palmitate, and cytokines were present throughout the incubation (a further 1‐h). For L6 experiments, cells were cultured and differentiated as described previously (Sinha et al. 2004). Subsequently, cells were exposed to the indicated conditions for 24‐h in the presence of 0.2 mmol L^−1^ 3‐[^3^H]‐palmitate.

### RNA extraction and qRT‐PCR

Total RNA from 30 to 200 mg of liver, skeletal muscle, or adipose tissue was extracted using Trizol reagent (Invitrogen, Carlsbad, CA) as described by the manufacturer. Subsequently, 1 *μ*g of RNA was used for a reverse transcriptase reaction using oligo(dT)_18_ primer (1^^ *μ*mol L^−1^), again as described by the manufacturer (Clontech, Mountain View, CA). Gene expression was measured using quantitative real‐time PCR. The abundance of mRNA was determined by comparative cycle threshold analysis using an ABI7900 (Applied Biosystems, Branchburg, NJ).

### Statistical analysis

Statistical significance was determined using, where applicable, repeated measures analysis of variance (RM‐ANOVA), the Kruskal–Wallis one‐way ANOVA, and unpaired *t*‐tests. All statistics were performed using the SPSS statistical program (Chicago, IL). All results are expressed as means ± SE. Significance was assumed at *P* < 0.05.

## Results

### p50^−/−^ mice have an elevated basal metabolic rate and increased skeletal muscle oxidative metabolism that is associated with decreased DGAT2 expression

**Figure 1:**
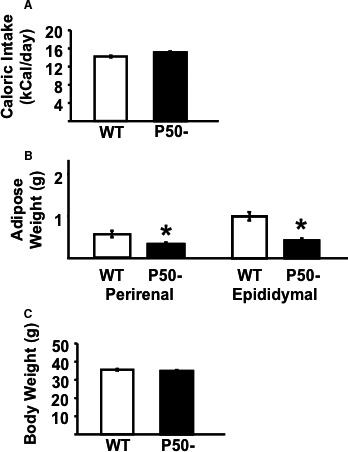
Body weight, caloric intake, and adiposity in p50^−/−^ and wild‐type mice. Caloric intake (Panel A), and adiposity (Panel B), and body weight (Panel C) were assessed in male p50 null mice (P50−) and their wild‐type littermate controls (WT). Results are presented as mean ± SE. *n* = a minimum of six animals for each group. Statistical significance is indicated (*P < 0.05 vs. corresponding control, unpaired *t*‐test).

**Figure 2:**
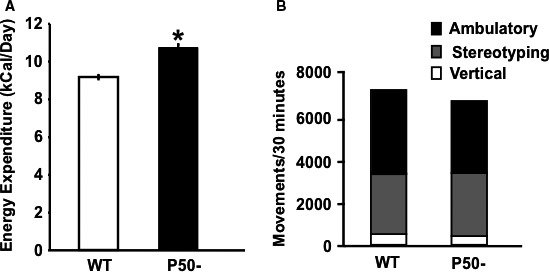
Total energy expenditure over hourly periods (Panel A) was measured in free moving animals using open‐circuit indirect calorimetry as described in Materials and Methods. Activity levels (Panel B) were measured using an open field test chamber. Results are presented as mean ± SE. *n* = a minimum of six animals for each group. Statistical significance is indicated (*P < 0.05 vs. WT, unpaired *t*‐tests)

We first assessed caloric intake, activity, metabolic rate, and oxidative metabolism in p50^−/−^ and littermate control mice, since alterations in these variables are associated with resistance to weight gain. Importantly, even in lean animals adiposity was significantly (~30%) decreased in p50^−/−^ compared to WT (Fig. 1, Panel B), but not to a level that decreased total (lean + fat) body weight (Fig. 1, Panel C), since lean mass accounts for approximately 75% of total weight in lean animals. Although caloric intake (Fig. 1, Panel A) was not statistically increased in p50^−/−^ mice on a standard chow diet there was a trend upwards (15.1 ± 0.23 vs. 14.3 ± 0.19 kCal/animal/day in p50^−/−^ and WT, respectively). Notably, energy expenditure was significantly greater (~20%) in p50^−/−^ compared to WT (Fig. 2, Panel A). These differences could not be explained by changes in activity levels, since ambulatory, stereotyping, and vertical movements were similar in both groups (Fig. 2, Panel B).

**Figure 3:**
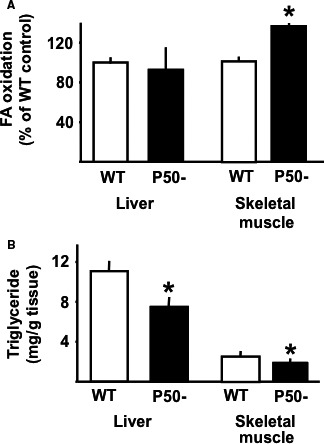
Fatty acid oxidation and triglycerides in skeletal muscle and liver of p50^−/−^ mice and wild‐type littermate controls. Panel A: Solei muscles and liver sections were isolated from overnight fasted P50− and WT mice. Subsequently, fatty acid oxidation was measured ex vivo as described in Materials and Methods. Results are presented as mean ± SE. *n* = 10 muscles or liver sections in each group. Statistical significance is indicated (*P < 0.05 vs. WT, unpaired *t*‐test). Panel B: Liver and gastrocnemius muscle were isolated from fasted P50− and WT. Subsequently, tissue triglyceride levels were measured as described in Materials and Methods. Results are presented as mean ± SE. *n* = a minimum of six animals for each group. Statistical significance is indicated (*P < 0.05 vs. corresponding control, unpaired *t*‐test).

**Figure 4:**
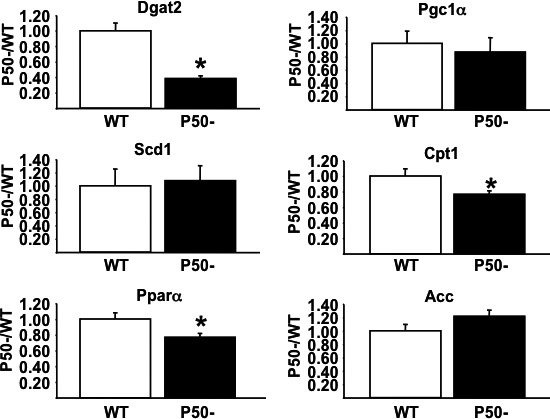
Skeletal muscle metabolic gene expression. qRT‐PCR was used to assess the expression of a number of skeletal muscle metabolic genes in P50− and WT mice as described in Materials and Methods. Results are presented as mean ± SE. *n* = a minimum of six animals for each group. Statistical significance is indicated (*P < 0.05 vs. corresponding control, unpaired *t*‐test). Primer sequences: ACC ‐ FOR 5′‐TGTTCTCGGCCTCTCTTCAC‐3′ REV 5′‐GAGGCTGCATTGAACACAAG‐3′; CPT1 *α* ‐ FOR 5′AGTGGCCTCACAGACTCCAG‐3′ REV 5′‐GCCCATGTTGTACAGCTTCC‐3′; DGAT2 ‐ FOR 5′‐GAAGATGTCTTGGAGGGCTG‐3′ REV 5′‐CGCAGCGAAAACAAGAATAA‐3′; PGC1 *α* ‐ FOR 5′‐TGAGGACCGCTAGCAAGTTT‐3′ REV 5′‐TGTAGCGACCAATCGGAAAT‐3′; PPAR *α* ‐ FOR 5′‐CAGTGGGGAGAGAGGACAGA‐3′ REV 5′‐AGTTCGGGAACAAGACGTTG‐3′; SCD1 ‐ FOR 5′‐CAGCCGAGCCTTGTAAGTTC‐3′ REV 5′‐GCTCTACACCTGCCTCTTCG‐3′.

The increase in metabolic rate in p50^−/−^ mice was associated with an ~35% increase in skeletal muscle, but not liver oxidative metabolism (Fig. 3, Panel A). Triglycerides in both skeletal muscle and liver were reduced (Fig. 3, Panel B), as were plasma triglycerides, but not FFA (Table 1). Interestingly, there was an ~70% decrease in skeletal muscle DGAT2 expression in p50^−/−^, suggesting a biochemical mechanism for reduced skeletal muscle triglycerides and elevated oxidative metabolism (Fig. 4). Surprisingly, however, the expression of two genes associated with the regulation of oxidative metabolism, PPAR*α* and CPT1, were reduced (Fig. 4), possibly pointing to a compensatory response.

**Figure 5:**
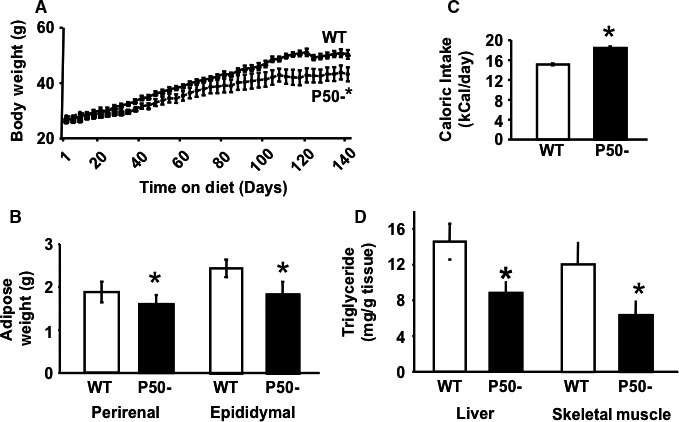
Body weight gain, caloric intake, adiposity, liver, and skeletal muscle triglyceride levels in p50^−/−^ mice in response to high‐fat diet exposure. p50 null mice (P50−) and their wild‐type littermate controls (WT) were placed on a high‐fat diet (45% calories from fat) as described in Materials and Methods. Body weight gain (Panel A) and caloric intake (Panel C) were measured biweekly over that time period. At the time of sacrifice, epididymal and perirenal adipose depots were excised and weighed (Panel B). Liver and skeletal muscle were isolated and assessed for triglyceride content (Panel D). *n* = a minimum of six animals for each group. Results are presented as mean ± SE. Statistical significance is indicated. *P < 0.05 (vs. WT, RM‐ANOVA for Panel A, Kruskal–Wallis one‐way ANOVA for Panels B and D, and unpaired *t*‐test for Panel B).

As previously reported (Gao et al. 2009), a high fat diet induced significant weight gain in both p50^−/−^ and WT (Fig. 5, Panel A). However, weight gain in p50^−/−^ was reduced by ~50% compared to WT, and this was matched (Fig. 5, panel B) by decreased adiposity (perirenal fat weight, 1.5 ± 0.24 g vs. 2.0 ± 0.24 g, and epididymal fat weight 1.8 ± 0.29 g vs. 2.6 ± 0.15 g, in p50^−/−^ compared to WT), despite significantly higher caloric intake (Fig. 5, Panel C) in p50 null mice (18.6 ± 0.35 kCal/animal/day in p50 null mice vs. 15.3 ± 0.20 kCal/animal/day in WT). Reduced adiposity was also reflected in the extent of liver steatosis, which was reduced in p50^−/−^ compared to WT (Fig. 5, Panel D). Furthermore, skeletal muscle triglyceride content, plasma triglycerides, and plasma free fatty acids were lower in p50^−/−^ (Fig. 5, Panel D and Table 2).

### Skeletal muscle, adipose tissue, and systemic IL‐6 and TNF*α* expression are increased in p50^−/−^ mice

**Figure 6:**
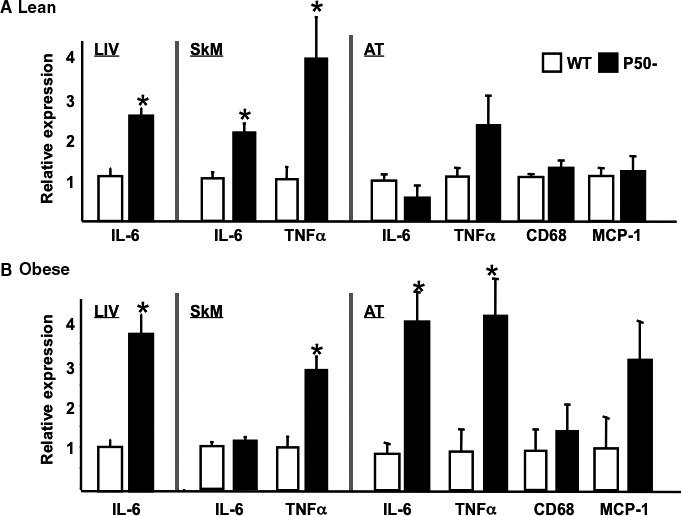
Inflammatory marker expression in liver, skeletal muscle, and adipose tissue of lean and obese p50^−/−^ and wild‐type mice. Quantitative real‐time PCR (qRT‐PCR) was used to assess the expression of a number of markers of inflammation in liver (LIV), skeletal muscle (SkM), and adipose tissue (AT) isolated from lean (Panel A) and obese (Panel B) p50− and WT mice, as detailed in Materials and Methods. Data are presented as mean ± SE. *n* = a minimum of six animals for each group. Statistical significance is indicated. *P < 0.05 (vs. corresponding WT control, unpaired *t*‐test). Primer sequences: CD68 ‐ FOR 5′‐TGACCTGCTCTCTCTAAGGCTACA‐3′ REV 5′‐TGGTCACGGTTGCAAGAGAA‐3′; IL‐6 ‐ FOR 5′‐CTGCAAGAGACTTCCATCCAGTT‐3′ REV 5′‐GAAGTAGGGAAGGCCGTGG‐3′; MCP‐1 ‐ FOR 5′‐TTCCTCCACCACCATGCAG‐3′ REV 5′‐CCAGCCGGCAACTGTGA‐3′; TNF *α* ‐ FOR 5′‐GGCACTCCCCCAAAAGATG‐3′ REV 5′‐GCCACAAGCAGGAATGAGAAG‐3′.

A number of cytokines and adipokines have been implicated in altering oxidative metabolism. To address the potential contribution of these mechanisms to the elevated oxidative metabolism in skeletal muscle observed in this study, we evaluated expression of a number of adipokines and cytokines implicated in the regulation of metabolism (Tables 1 and 2, and Fig. 6). Plasma concentration of adiponectin and resistan were similar in WT and p50^−/−^ lean and obese mice (Tables 1 and 2). Plasma leptin was similar in lean p50^−/−^ and WT and, as would be expected, was increased in obesity in both WT and p50^−/−^ animals. However, the absolute increase was less in p50^−/−^, most likely reflecting the reduced adiposity in these mice. Together, these data suggest that alterations in adipokine concentrations do not account for the elevation in oxidative metabolism in p50^−/−^ mice.

**Table 1:** Plasma metabolic variables of lean p50− and lean wild‐type mice

	WT	P50−
FFA (mmol/L)	0.51 ± 0.07	0.37 ± 0.03
Triglycerides (mg/dL)	30.9 ± 2.7	21.3 ± 2.8*
Glucose (mmol/L)	9.8 ± 0.7	8.4 ± 1.1
Insulin (*μ*IU/mL)	7.2 ± 1.2	7.7 ± 2.4
HOMA	176 ± 2	162 ± 7
Leptin (ng/mL)	2.8 ± 0.8	1.3 ± 0.5
Adiponectin (*μ*g/mL)	9.0 ± 0.5	9.8 ± 0.5
Resistin (ng/mL)	2.3 ± 0.3	2.2 ± 0.3
IL‐6 (pg/mL)	11.0 ± 1.8	28.5 ± 7.8*
TNF*α* (pg/mL)	ND	ND

Wild‐type and P50^−/−^ mice were fasted and blood samples taken, and plasma concentrations of indicated metabolites, hormones, and cytokines were assessed as detailed in Materials and Methods. *n* = a minimum of six animals. Statistical significance is indicated (*P < 0.05). ND = not detectable.

**Table 2:** Plasma metabolic variables of obese p50− and obese wild‐type mice

	WT	P50−
FFA (mmol/L)	0.59 ± 0.06	0.45 ± 0.04*
Triglycerides (mg/dL)	36.6 ± 5.9	24.4 ± 2.6*
Glucose (mmol/L)	9.7 ± 1.0	9.8 ± 0.8
Insulin (*μ*IU/mL)	19.9 ± 5.6	21.1 ± 1.3
HOMA	485 ± 14	517 ± 3
Leptin (ng/mL)	30.7 ± 4.4	19.2 ± 5.2*
Adiponectin (*μ*g/mL)	12.8 ± 1.7	9.2 ± 1.8
Resistin (ng/mL)	4.3 ± 0.7	3.6 ± 0.5
IL‐6 (pg/mL)	24.9 ± 4.1	33.0 ± 6.2
TNF*α* (pg/mL)	ND	ND

Obese wild‐type and P50 mice were fasted overnight and blood samples taken, and plasma concentrations of indicated metabolites, hormones, and cytokines were assessed as detailed in Materials and Methods. *n* = a minimum of six animals. Statistical significance is indicated (*P < 0.05). ND = not detectable.

We next assessed IL‐6 and TNF*α* expression. The most striking difference between WT and p50^−/−^ was observed for plasma IL‐6 concentrations. Thus, plasma IL‐6 in lean p50^−/−^ was substantially greater than in lean WT (Table 1). Furthermore, while obesity increased IL‐6 in WT, this increase did not occur in p50^−/−^ (Table 2). Plasma TNF*α* was not detectable under any of the conditions tested. In skeletal muscle, IL‐6 and TNF*α* mRNA were elevated in lean p50^−/−^ compared to lean WT (Fig. 6, Panel A). In obese animals (Fig. 6, Panel B), the increased expression of TNF*α* in p50^−/−^ was maintained but IL‐6 expression was similar in obese p50^−/−^ and WT, a situation similar to that observed for plasma IL‐6 levels. In liver, TNF*α* was not detectable, but IL‐6 expression was elevated in both lean and obese p50^−/−^ compared to corresponding WT controls, as reported by Gao et al. (2009)). In adipose tissue, IL‐6, TNF*α*, CD68, and MCP1 mRNA levels were similar in lean p50^−/−^ and lean WT. However, in obesity both IL‐6 and TNF*α* expression were greater in p50^−/−^ compared to WT, but there were no differences in CD68 or MCP1 expression. Together, these data demonstrate that lean p50^−/−^ mice have elevated expression of cytokines in blood, liver, and skeletal muscle, but not in adipose tissue. Obesity normalizes systemic and skeletal muscle levels of IL‐6 between p50^−/−^ and WT, but elevated TNF*α* (skeletal muscle and adipose tissue) and IL‐6 (liver and adipose tissue) expression is maintained.

**Figure 7:**
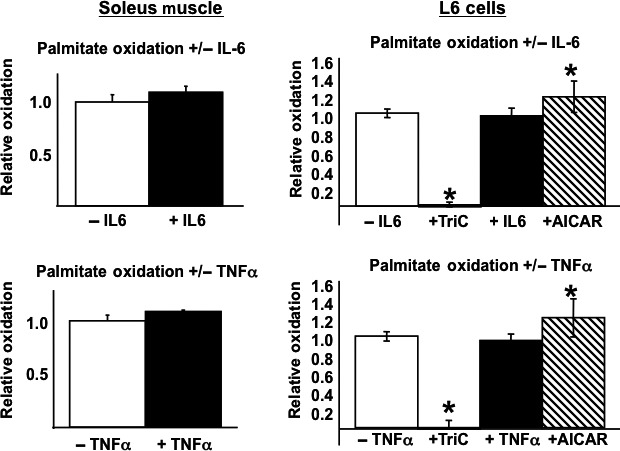
The effects of IL‐6 and TNF*α* on skeletal muscle fatty acid oxidation. L6 myotubes or isolated soleus muscles from WT mice were exposed to IL‐6 or TNF*α* for 24‐h (L6 cells) or 2‐h (soleus muscle) in the presence of palmitate for the measurement of fatty acid oxidation as described in Materials and Methods. Additionally, L6 myotubes were exposed to AICAR, the AMPK activator, or Triacsin C, the Acyl Co‐A inhibitor. Data are presented as mean ± SE. *n* = a minimum of four independent experiments for each condition. Statistical significance is indicated. *P < 0.05 (vs. corresponding control, unpaired *t*‐test for soleus muscle, Kruskal–Wallis one‐way ANOVA for L6 cells).

We next determined the potential contribution of skeletal muscle cytokine gene expression alterations to the skeletal muscle phenotype of these animals, that is, elevated oxidative metabolism. To address this issue fatty acid oxidation was assessed in isolated solei muscles and L6 myotubes exposed to TNF*α* or IL‐6. As Figure 7 shows, neither cytokine altered oxidative metabolism in either soleus muscle or L6 cells. Importantly, under similar experimental conditions, the fatty acyl‐CoA sythethase inhibitor Triacsin C blocked, whereas the AMPK activator AICAR increased, fatty acid oxidation in L6 cells.

## Discussion

The major novel observation arising from this study is that increased metabolic rate contributes to the decreased weight in mice lacking the p50 subunit of NF‐*κ*B. A major contributing mechanism of elevated metabolic rate is an increase in skeletal muscle oxidative metabolism, resulting in reduced skeletal muscle and systemic lipid levels. The mechanisms driving increased skeletal muscle oxidative metabolism and reduced lipid levels are not categorically identified, but may be related to a substantial decrease in skeletal muscle DGAT2 expression. Finally, although the cytokines IL‐6 and TNF*α* are elevated in skeletal muscle of p50 null mice, we were unable to demonstrate a role for either cytokine in mediating alterations in muscle oxidative metabolism.

Altered caloric intake or energy expenditure (metabolic rate) are the two most common variables effecting body composition. In this study, differences in total body weight were not evident in standard chow fed animals, but there was a small reduction in adiposity, and this difference between p50^−/−^ and WT became more pronounced when animals were challenged with a high‐fat diet. The reduced body weight gain on a high‐fat diet is even more striking when it is considered that caloric intake was significantly higher in p50^−/−^ animals compared to controls. It remains unclear why no differences in caloric intake were evident on a standard chow diet, although there was a trend toward an increase, which again rules out caloric intake as a contributory mechanism to decreased adiposity. On the basis of these observations, we hypothesized that metabolic rate should be increased in the p50^−/−^ animals. Indeed, this proved to be case. Energy expenditure over a 24 h period was elevated by ~20% in p50^−/−^ animals. Notably, the increase was present throughout the 24 h period measured (data not shown). The increase in metabolic rate could also not be attributed to changes in different aspects of activity, since these were all similar in null and wild‐type animals.

An elevated metabolic rate in the absence of alterations in activity, taken with the generalized decrease in tissue and plasma lipids suggested to us that overall oxidative metabolism was elevated in p50^−/−^ mice. Since skeletal muscle comprises by mass the largest oxidative tissue in the body, we thought it likely that this tissue contributed to the overall phenotype. This proved to the case, and likely explains a large proportion of the whole‐body increase in metabolic rate. Of interest, the increase in skeletal muscle fatty acid oxidation (~35%) was somewhat greater than the increase in overall metabolic rate (~20%) perhaps reflecting the tissue specificity of this effect. It would be expected that the increase in whole‐body metabolic rate would be less than that in a specific tissue if oxidative metabolism were not increased in all tissues. However, it should also be noted that other mechanisms that were not assessed in our study may also contribute to the increase in metabolic rate/reduced weight gain on the high‐fat diet. Specifically, an increase in thermogenesis mediated by brown adipose tissue (BAT) amount or activity could play a role. Furthermore, we did not compare the efficiency of caloric extraction in our models. A reduced caloric extraction in the p50 null mice would result in a reduction in weight gain compared to WT, and would manifest as increased p50 null fecal carbon content.

A gene expression analysis identified changes in the expression of a number of skeletal muscle genes involved in the regulation of lipid and oxidative metabolism. Most notably, DGAT2 expression was decreased by ~70%. DGAT2 catalyzes the final step in triglyceride synthesis, and is considered the major skeletal muscle DGAT isoform. Notably, deletion of DGAT1 increases metabolic rate and oxidative metabolism and reduces weight gain on a high‐fat diet (Smith et al. 2000). However, the metabolic effects of DGAT2 deletion remain unknown. Surprisingly, given the increases in oxidative metabolism, skeletal muscle CPT1, and PPAR*α* gene expression was reduced. While the mechanisms of these changes remain unknown, it could be speculated that the decreases are an attempted compensatory response to inappropriately elevated oxidative metabolism.

Our data demonstrate that p50^−/−^ mice have elevated expression of the inflammatory cytokines IL‐6 and TNF*α* in blood (IL‐6), liver (IL‐6), and skeletal muscle (IL‐6 and TNF*α*). TNF*α* induces insulin resistance (Hotamisligil et al. 1993; Hotamisligil 1999; Moller 2000), has been implicated as important mediator of cachexia, and a role for TNF*α* in increased fatty acid oxidation has been proposed, but data are inconclusive in this regard (Bruce and Dyck 2004). It has also been reported that IL‐6 increases fatty acid oxidation in L6 myotubes (Petersen et al. 2005) and in isolated rat skeletal muscle (Bruce and Dyck 2004), whereas a low‐dose infusion of IL‐6 for 1 h in humans increases fatty acid oxidation (van Hall et al. 2003). In the context of body weight regulation, Metzger et al. (2001) reported that chronic elevation of IL‐6 leads to a significant loss of fat tissue without any effect on lean body mass, whereas Wallenius et al. (2002) demonstrated that IL‐6 knockout mice become obese with age and have impaired glucose tolerance, whereas replacement of IL‐6 in the knockout mice leads to weight loss. Notably, these observations fit with the reduced adiposity and elevated skeletal muscle fatty acid oxidation in p50^−/−^ mice observed in this study, and are consistent with the hypothesis that elevations in IL‐6, and possibly TNF*α*, play a role in mediating the alterations in body composition and oxidative metabolism. However, in our hands, neither cytokine increased fatty acid oxidation in either isolated soleus muscle or L6 myotubes. It is possible that the length of exposures was insufficient (2‐h for soleus muscle and 24‐h for L6 myotubes), although these exposure times are similar to those used in previous in vitro studies, and the models (in our hands) are not suitable for longer exposures. In short, our data do not rule out a role for IL‐6 or TNF*α* in the skeletal muscle phenotype, but addressing this issue definitively will require more sophisticated approaches, such as the crossing of p50 null mice with IL‐6 (TNF*α*) null mice.

It is notable that deletion of a NF‐*κ*B subunit resulted in an increase in the expression of genes regulated by the NF‐*κ*B pathway. These data suggest that deletion of the p50 subunit has the effect of increasing overall activity of the NF‐*κ*B pathway. However, we were unable to directly address this hypothesis in vivo, with the exception that we observed no alterations in I*κ*B*α* levels between p50^−/−^ and WT mice (data not shown). It is worthwhile, nonetheless, to speculate on these observations. In this regard, p50 acts as a stimulator and inhibitor of NF‐*κ*B pathway activity depending on cell type (Beinke and Ley 2004; Gadjeva et al. 2004), suggesting that deletion of p50 in muscle, liver, and/or adipose tissue may remove an inhibitory regulator. It is also possible that compensatory mechanisms, such as the partnering of p65 with other NF‐*κ*B subunits in the absence of p50 may explain the increased inflammatory phenotype.

It has been proposed that inhibitors of activity of the NF‐*κ*B pathway may prove useful in the treatment of insulin resistance. Indeed, there are clear beneficial effects on insulin action of the IKK inhibitor salicylate (Yuan et al. 2001; Hundal et al. 2002), and salsalate, a chemical derivative of salicylic acid has shown some promise in a small clinical trial (Fleischman et al. 2008). Furthermore, there are many modulators of activity of the NF‐*κ*B pathway that have been developed for their potential use in the treatment of inflammatory disorders such as rheumatoid arthritis and inflammatory bowel disease (Epinat and Gilmore 1999; Verma 2004; Pande and Ramos 2005). However, issues relating to tissue specificity, side‐effects on immune system activity and unanticipated effects on metabolism of these compounds are possible confounders in their use in the treatment of insulin resistance. In this regard, our study strikes a cautionary note. An intervention that targeted the inactivation of a NF‐*κ*B subunit implicated in the pathogenesis of the metabolic abnormalities resulted in a metabolic profile, which although beneficial in some ways (reduced weight, decreased adiposity, improved lipid profiles), was possibly detrimental (elevated expression of inflammatory cytokines) in other ways. Thus, further studies addressing the role of individual elements of the NF‐*κ*B pathway in the pathogenesis of metabolic abnormalities are necessary and will likely clarify these issues.

## Conflict of Interest

The authors have no conflicts.
